# Muscle weakness is more strongly associated with functional outcomes in patients with stroke than sarcopenia or muscle wasting: an observational study

**DOI:** 10.1007/s40520-023-02672-9

**Published:** 2024-01-23

**Authors:** Masafumi Nozoe, Hiroki Kubo, Miho Yamamoto, Rio Ikeji, Haruka Seike, Kazuki Majima, Shinichi Shimada

**Affiliations:** 1 Department of Physical Therapy, Faculty of Rehabilitation, Kansai Medical University, 18-89 Uyamahigashicho, Hirakata, Osaka Japan; 2 Department of Physical Therapy, Faculty of Nursing and Rehabilitation, Konan Women’s University, Kobe, Japan; 3 Department of Rehabilitation Medicine, Nishiyamato Rehabilitation Hospital, Nara, Japan; 4 Department of Rehabilitation, Itami Kousei Neurosurgical Hospital, Itami, Japan; 5 Department of Neurosurgery, Itami Kousei Neurosurgical Hospital, Itami, Japan

**Keywords:** Sarcopenia, Muscle weakness, Muscle mass, Modified Rankin Scale, Sarcopenia assessment tool

## Abstract

**Background:**

Stroke-related sarcopenia is an important prognosis factor and an intervention target for improving outcomes in patients with stroke.

**Aim:**

This study aimed to identify the association between sarcopenia, possible sarcopenia, muscle weakness, muscle mass and calf circumference, and the functional outcomes 3 months after stroke.

**Methods:**

In this single-centre prospective observational study, muscle strength, muscle mass, and calf circumference were measured in patients with acute stroke at hospital discharge. Diagnosis of sarcopenia, possible sarcopenia, muscle weakness, low muscle mass, and low calf circumference were defined according to the 2019 Asian Working Group for Sarcopenia criteria. The primary outcome measure was the modified Rankin Scale (mRS) score at 3 months, with an mRS score of 3 or higher indicating a poor outcome. Logistic regression analysis was conducted to examine independent associations between each assessment and functional outcomes.

**Results:**

A total of 247 patients (median age: 73 years) were included in this study. The prevalence of sarcopenia was 28% (*n* = 70), and in the adjusted model, sarcopenia (aOR = 2.60, 95% CI 1.07–6.31, *p* = 0.034), muscle weakness (aOR = 3.40, 95% CI 1.36–8.52, *p* = 0.009), and low muscle mass (aOR = 2.61, 95% CI 1.04–6.52) were significantly associated with poor functional outcome. Nevertheless, other evaluations did not demonstrate an independent association with the outcome.

**Conclusion:**

Sarcopenia, muscle weakness, and low muscle mass were found to be independently associated with functional outcomes 3 months after stroke, and muscle weakness exhibited the strongest association with outcomes among them.

## Introduction

Stroke is a prominent global prognostic factor [1], which frequently leads to disabilities [2]. Numerous factors have been identified that play a role in the development of disability in patients with stroke, such as age [3], stroke severity [4], comorbidities [5], premorbid functional status [6], or stroke type [7] and location [8]. In addition to these potential factors, the effects of interventions such as recanalization [9], early mobilisation [10], and post-stroke conditions, such as the incidence of stroke-related complications [11], also affect the functional outcomes in these patients.

Sarcopenia, a condition characterised by a decline in muscle mass and function, has gained significant recognition owing to its impact on disability [12]. This condition is typically associated with aging and other factors, including malnutrition, sedentary lifestyle, illness, and hospitalisation [13]. Stroke often causes hemiplegia, spasticity, deconditioning, neuropathic pain, and reduced physical activity [14]. Furthermore, it is considered a contributing factor to the development of sarcopenia, often referred to as stroke-related sarcopenia (SRS) [15, 16]. SRS is an important prognosis factor [17, 18] and it is also recognised as an intervention target for improving outcomes in those patients [19]. Moreover, it is worth noting that the prevalence of SRS tends to increase during the acute phase of stroke [16, 20]. This highlights the importance of implementing early and efficient SRS assessments to promptly identify and address these issues. The development of practical and reliable assessment tools that can be readily applied in acute stroke settings is beneficial for optimising patient care and improving outcomes.

The assessment of sarcopenia according to the 2019 Asian Working Group for Sarcopenia (AWGS2019) criteria involves two main components: case findings and diagnosis [12]. These assessments included measurements of the calf circumference (CC), muscle strength, and muscle mass [12]. Based on the results obtained from these assessments, individuals can be categorised as having possible or diagnosed sarcopenia [12]. In the clinical setting, it is important to have assessments that are easy to perform and convenient. Consequently, numerous studies have attempted to estimate sarcopenia or appendicular muscle mass using muscle strength, CC, questionnaires, and combinations thereof [21–26]. CC measurement can be useful for evaluating sarcopenia and as a prognostic factor in patients with stroke [27–32]. Currently, only one study has assessed the relationship between these assessments and short-term functional outcomes assessed at hospital discharge in patients with stroke [29]. Given that the functional outcome at 3 months after stroke is a critical indicator for assessing the overall recovery and prognosis of stroke patients [31, 32], it is important to investigate the influence of muscle mass, muscle strength, and CC, in addition to diagnosed sarcopenia or possible sarcopenia on this specific outcome. This information will greatly contribute to the development of comprehensive assessment protocols for stroke patients, enabling more accurate prognoses and targeted interventions.

The main aim of this study was to investigate the relationship between sarcopenia, possible sarcopenia, muscle mass, muscle strength, and CC, and the functional outcomes observed 3 months post-stroke.

## Methods

### Participants

This study enrolled patients with stroke who were admitted to a single institution within 48 h of stroke onset between June 2020 and March 2022. To be eligible for inclusion, patients were aged < 85 years and had a premorbid modified Rankin Scale (mRS) [32] score of 0 or 1, indicating no disability. Patients with subarachnoid haemorrhage, the presence of pitting oedema [27], impaired consciousness, cognitive dysfunction, language disorders, or death during hospitalisation were excluded from the study. All participants underwent rehabilitation, which included early mobilization, physical therapy, and occupational therapy. Sessions typically lasted between 20 and 60 min daily. The primary goal of rehab was to enhance physical capability and daily tasks. Each patient’s plan was tailored by their physician before discharge. Before participating in the study, all participants provided informed consent. The study was approved by the Institutional Review Board of Konan Women’s University (approval number: 2019030).

### Baseline characteristic

Baseline characteristic data were collected from the patients’ electronic medical records. The collected patient characteristics included age, sex, National Institutes of Health Stroke Scale (NIHSS) score upon admission [4], stroke type and side, comorbidities (previous stroke, hypertension, diabetes mellitus, hypercholesterolaemia, congestive heart failure, or atrial fibrillation), recanalization therapy (thrombolysis or thrombectomy), rehabilitation time (daily duration of physical and occupational therapy during the first week from admission), length of hospital stay, discharge destination (home discharge), and mRS score at discharge. The mRS score was used to evaluate the level of disability or dependence in daily activities at discharge. The mRS score ranges from 0 (no symptoms) to 6 (death), with higher scores indicating greater disability [32].

### Measurements for body composition, muscle strength, and calf circumference

Body composition, muscle strength, and CC were measured at hospital discharge. Muscle mass was measured using body impedance analysis (BIA) while the patients were in the supine position, specifically using InBody S10 equipment (InBody, Tokyo, Japan) [27]. These measurements were taken three hours after lunch to minimise the potential effects of recent food intake. Appendicular muscle mass was estimated from the appendicular lean mass obtained using BIA measurements [33]. To assess the relative muscle mass, the skeletal muscle index (SMI) was calculated by dividing the measured appendicular muscle mass by the square of the patient’s height (m^2^).

To evaluate muscle strength, hand grip (HG) strength was measured on the non-paretic side of patients with paralysis using a Smedley dynamometer (model 103-S; Hata Sporting Goods Industries Ltd., Tokyo, Japan). In cases without paralysis, HG strength was measured on both sides, and the highest value was recorded. For each participant, three measurements were performed in either a standing or seated position with the arms positioned straight on the sides [33]. The maximum recorded value was used for the analysis.

CC was measured on the non-paretic side in patients with paralysis. These measurements were performed following the muscle mass measurements. CC measurements were taken while the patients were in the supine position with their knee joints flexed at a 90° angle. The feet and ankles were relaxed during the measurement. A flexible tape was wrapped perpendicularly around the axis of the leg, and the maximum CC was recorded in increments of 0.1 cm. In cases without paralysis, measurements were taken for both legs, and the highest value was recorded [27].

### Criteria for sarcopenia, possible sarcopenia, and other assessments

In this study, the assessment and diagnosis of sarcopenia followed the criteria outlined by the AWGS2019 [12]. Low SMI was determined using specific cutoff values of < 7.0 kg/m^2^ for men and < 5.7 kg/m^2^ for women [12]. Muscle weakness was defined as reduced HG strength, with cutoff values at < 28 kg for men and < 18 kg for women. To diagnose sarcopenia, both a low SMI and muscle weakness, as determined by HG strength, are required. This approach was aligned with established guidelines [12]. Additionally, low CC was assessed using specific cutoff values of < 34 cm for men and < 33 cm for women [12]. To identify possible sarcopenia, the presence of low CC and muscle weakness is required [12].

### Outcomes

The primary outcome measure in this study was the mRS score, which was assessed either from medical records or through mail evaluation conducted 3 months after the stroke. A poor outcome was defined as an mRS score of 3 or higher at the 3-month follow-up [34], given that this metric is widely recognized as the primary method for evaluating stroke outcomes [31, 32].

### Sample size calculation

Based on a previous study on the prevalence of pre-stroke sarcopenia and its association with poor outcomes 3 months after stroke, a sample size calculation was performed [35]. In the previous study, the prevalence of sarcopenia was 18%, the proportion of poor outcomes in patients with sarcopenia was 50%, and the proportion of poor outcomes in non-sarcopenic patients was 12% [35]. The calculation was conducted to determine the minimum number of participants required to achieve a statistical power of 0.95 and an alpha error of 0.05. The sample size was calculated using G*Power version 3.1.9.6, developed by Heinrich-Heine-Universität Düsseldorf, Germany. The calculated sample size indicated that minimum 112 participants were required to achieve the desired statistical power and significance.

### Statistical analysis

The results are presented as medians and interquartile ranges (IQRs) for continuous variables, whereas categorical variables were reported as numbers and percentages. Patients were divided into two groups based on their sarcopenia diagnosis: non-sarcopenia and sarcopenia. Appropriate statistical tests were used to compare the baseline characteristics, sarcopenia assessment, and outcomes between the two groups. The Mann–Whitney *U* test was used for continuous variables, whereas the Pearson *χ*^2^ test and Fisher’s exact test were used for categorical variables. Adjusted odds ratios for sarcopenia, possible sarcopenia, muscle weakness, low SMI, and low CC were derived using logistic regression to assess their association with poor functional outcomes, defined as an mRS score of 3–6. The odds ratios were adjusted for potential confounding variables, including age, sex, length of hospital stay, NIHSS score, previous stroke, and rehabilitation time. All statistical analyses were performed using the EZR software package, which provides a graphical user interface for R (version 1.55). A statistical significance level of *p* < 0.05 was considered to indicate a significant association or difference between groups.

## Results

Of the 667 patients with stroke admitted to the hospital during the study period, 157 were excluded because of their age (≥ 85 years), 98 had premorbid dependency (mRS ≥ 2), and 59 were admitted more than 48 h after stroke onset. Additionally, 4 patients died during hospitalisation, 26 were discharged early, and 17 had missing data.

Among the remaining 306 participants, 10 participants were excluded because of a lack of measurements caused by the COVID-19 pandemic, 1 participant because of lower limb amputation that hindered the measurement of SMI, 3 participants because of a lack of informed consent resulting from psychiatric disorders, 4 participants declined to participate, and 41 participants were lost to follow-up. Finally, 247 patients were included in the final analysis.

Table 1 presents the baseline characteristics of patients in each group. Among the 247 patients included in the analysis, 70 (28%) were diagnosed with sarcopenia at hospital discharge. The median age of the patients was 73 years (IQR: 65–78), and 82 patients (33%) were female. The median NIHSS score was 3 (IQR: 1–5), and the median length of hospital stay was 15 days (IQR: 9–23).

**Table 1:** Baseline characteristics of patients with sarcopenia and without sarcopenia (non-sarcopenia)

	Total (*n* = 247)	Non-sarcopenia (*n* = 177)	Sarcopenia (*n* = 70)	*p*-value
Age (years, median (IQR))	73 (65–78)	71 (64–76)	77 (72–79)	< 0.001
Sex (male/female)	165/82	123/54	42/28	0.178
BMI (kg/m^2^, median (IQR))	23.8 (21.4–26.3)	24.9 (22.7–27.2)	21.3 (19.9–23.2)	< 0.001
NIHSS score (median (IQR))	3 (1–5)	2 (1–4)	5 (2–9)	< 0.001
Stroke type, *n* (%)				
Infarction	206 (83)	153 (86)	53 (76)	0.057
Haemorrhage	41 (17)	24 (14)	17 (24)	
Side of lesion (right/left/both)	120/124/3	90/84/3	30/40/0	0.338
Comorbidities (%)				
Previous stroke	50 (20)	29 (16)	21 (30)	0.022
Hypertension	156 (63)	112 (63)	44 (63)	1.000
Diabetes mellitus	53 (21)	35 (20)	18 (26)	0.307
Hypercholesterolemia	106 (43)	78 (44)	28 (40)	0.570
Congestive heart failure	3 (1)	2 (1)	1 (1)	1.000
Atrial fibrillation	31 (13)	17 (10)	14 (20)	0.033
Recanalization therapy (%)				
Thrombolysis	7 (3)	6 (3)	1 (1)	0.677
Thrombectomy	8 (3)	5 (3)	3 (4)	0.691
Rehabilitation time (min/day)				
Physical therapy	39 (31–49)	40 (31–46)	43 (31–46)	0.307
Occupational therapy	41 (31–46)	40 (31–49)	40 (34–51)	0.862
Length of hospital stay (days, median (IQR))	15 (9–23)	13 (9–20)	20 (15–25)	< 0.001
Home discharge (number (%))	157 (64)	130 (73)	27 (39)	< 0.001
mRS at discharge (%)				
0	49 (20)	44 (25)	5 (7)	< 0.001
1	58 (23)	48 (27)	10 (14)	
2	33 (13)	28 (16)	5 (7)	
3	41 (17)	25 (14)	16 (23)	
4	52 (21)	30 (17)	22 (32)	
5	14 (6)	2 (1)	12 (17)	

Rehabilitation time, daily duration of physical and occupational therapy during the first week from admission

*IQR* Interquartile range; *BMI* body mass index; *NIHSS* National Institute of Health Stroke Scale; *mRS* modified Rankin Scale

**Fig. 1:**
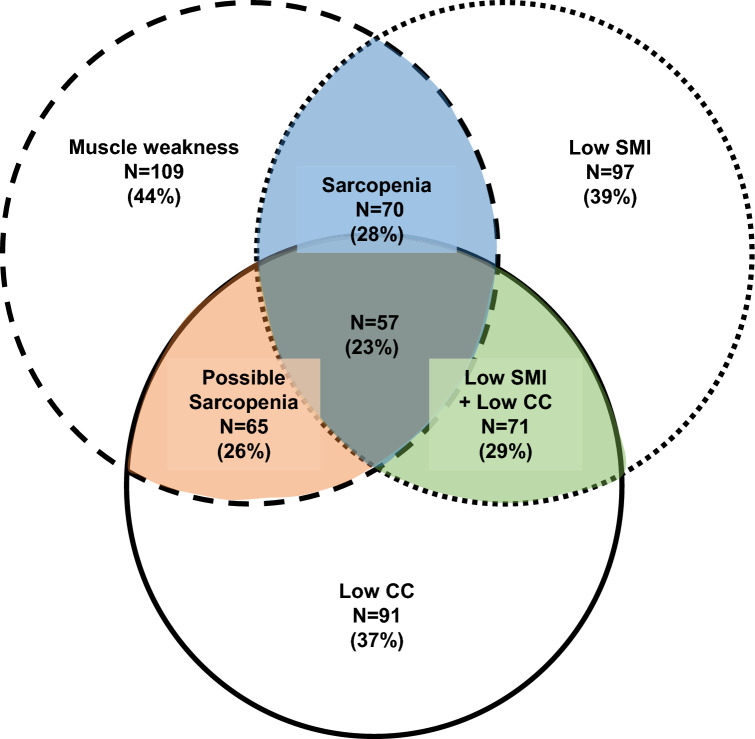
Prevalence of each sarcopenia components and the coexistence of these conditions. The figure shows three delineated sections: those enclosed by dashed-lines represent all individuals with muscle weakness (*n* = 109, 44%), those enclosed by dotted-lines represent all with low skeletal muscle mass (*n* = 97, 39%), and those bounded by solid lines represent all with decreased calf circumference (*n* = 91, 37%). The blue and grey portion indicates those diagnosed with sarcopenia (*n* = 70, 28%), the orange and grey portion represents possible sarcopenia (*n* = 65, 26%), and the green and grey portion indicates those with both low skeletal muscle mass and decreased calf circumference (*n* = 71, 29%). The grey portion encompasses individuals who fall below the cutoff values in all assessments (*n* = 57, 23%)

Table 2 displays the results of the sarcopenia assessments and outcomes for each patient group. All assessments showed significant differences between the groups (*p* < 0.001). Additionally, there was a significant difference in functional outcomes, as assessed by the mRS score, 3 months after the stroke (*p* < 0.001). Figure 1 shows the prevalence of various assessments, including diagnosed sarcopenia, possible sarcopenia, muscle weakness, low muscle mass, low CC, and the coexistence of these conditions. Among the study population, 148 (60%) patients were eligible for at least one sarcopenia assessment. This study included 109 patients with muscle weakness (44%), 97 with a low SMI (39%), and 91 with a low CC (37%).

**Table 2:** Assessment of sarcopenia and functional outcomes in patients with sarcopenia and without sarcopenia (non-sarcopenia)

	Total (*n* = 247)	Non-sarcopenia (*n* = 177)	Sarcopenia (*n* = 70)	*p*-value
Hand grip strength (kg, median (IQR))	27 (18–34)	30 (21–36)	17 (13–24)	< 0.001
Muscle weakness (number (%))	109 (44)	39 (22)	70 (100)	< 0.001
Calf circumference (cm, median (IQR))	34.5 (32.5–37.0)	35.5 (34.0–38.0)	31.5 (30.0–33.0)	< 0.001
Low calf circumference (number (%))	91 (37)	34 (19)	57 (81)	< 0.001
Possible sarcopenia (number (%))	65 (26)	8 (5)	57 (81)	< 0.001
SMI (kg/m^2^, median (IQR))	6.8 (5.9–7.5)	7.1 (6.5–7.9)	5.8 (5.0–6.4)	< 0.001
Low SMI (number (%))	97 (39)	27 (15)	70 (100)	< 0.001
mRS at 3 months after stroke (%)				
0	41 (17)	37 (21)	4 (6)	< 0.001
1	72 (29)	61 (34)	11 (16)	
2	75 (30)	54 (30)	21 (30)	
3	30 (12)	16 (9)	14 (20)	
4	23 (9)	7 (4)	16 (23)	
5	4 (2)	1 (1)	3 (4)	
6	2 (1)	1 (1)	1 (1)	

*IQR* Interquartile range; *SMI* skeletal muscle mass index; *mRS* modified Rankin Scale

Table 3 presents the logistic regression analysis of each assessment for poor outcomes 3 months after the stroke. In the crude model, all assessments were significantly associated with poor outcomes (*p* < 0.001). However, in the adjusted model, sarcopenia (aOR = 2.60, 95% CI 1.07–6.31, *p* = 0.034), muscle weakness (aOR = 3.40, 95% CI 1.36–8.52, *p* = 0.009), and low muscle mass (aOR = 2.61, 95% CI 1.04–6.52) remained independently associated with poor outcomes. Possible sarcopenia (aOR: 1.92; 95% CI 0.78–4.72; *p* = 0.154), and Low CC (aOR: 1.99; 95% CI 0.83–4.76; *p* = 0.123) were not significantly associated with poor outcomes in the adjusted model.

**Table 3:** Logistic regression analysis of poor outcomes 3 months after stroke

	Crude model		Sarcopenia model		Possible Sarcopenia model		Muscle weakness model		Low SMI model		Low CC model	
	OR (95% CI)	*p*-value	aOR (95% CI)	*p*-value	aOR (95% CI)	*p*-value	aOR (95% CI)	*p*-value	aOR (95% CI)	*p*-value	aOR (95% CI)	*p*-value
Age	1.03 (1.00–1.07)	0.0496	1.06 (1.00–1.12)	0.043	1.07 (1.01–1.13)	0.016	1.06 (1.00–1.1)	0.050	1.06 (0.99–1.12)	0.054	1.07 (1.01–1.13)	0.015
Sex (female)	2.04 (1.12–3.72)	0.020	1.69 (0.74–3.89)	0.214	1.51 (0.66–3.44)	0.330	1.36 (0.59–3.16)	0.470	1.93 (0.82–4.50)	0.130	1.64 (0.72–3.74)	0.239
NIHSS	1.48 (1.33–1.65)	< 0.001	1.37 (1.22–1.55)	< 0.001	1.38 (1.22–1.56)	< 0.001	1.38 (1.22–1.56)	< 0.001	1.38 (1.23–1.56)	< 0.001	1.38 (1.22–1.55)	< 0.001
Rehabilitation time	1.01 (0.997–1.02)	0.127	1.01 (0.99–1.03)	0.381	1.01 (0.99–1.03)	0.392	1.01 (0.99–1.03)	0.352	1.01 (0.99–1.03)	0.329	1.01 (0.99–1.03)	0.331
LOS	1.14 (1.09–1.18)	< 0.001	1.08 (1.02–1.14)	0.005	1.08 (1.02–1.14)	0.005	1.08 (1.02–1.14)	0.005	1.08 (1.02–1.14)	0.004	1.08 (1.02–1.14)	0.004
Previous stroke	2.14 (1.09–4.19)	0.027	0.93 (0.33–2.64)	0.886	1.04 (0.37–2.93)	0.945	0.94 (0.33–2.71)	0.922	0.95 (0.33–2.69)	0.919	1.11 (0.39–3.15)	0.847
Sarcopenia	5.74 (3.05–10.80)	< 0.001	2.60 (1.07–6.31)	0.034	–	–	–	–	–	–	–	–
Possible Sarcopenia	5.01 (2.67–9.43)	< 0.001	–	–	1.92 (0.78–4.72)	0.154	–	–	–	–	–	–
Muscle weakness	6.23 (3.18–12.20)	< 0.001	–	–	–	–	3.40 (1.36–8.52)	0.009	–	–	–	–
Low SMI	3.96 (2.14–7.32)	< 0.001	–	–	–	–	–	–	2.61 (1.04 –6.52)	0.041	–	–
Low CC	3.78 (2.06–6.97)	< 0.001	–	–	–	–	–	–	–	–	1.99 (0.83–4.76)	0.123

*SMI* Skeletal muscle mass index; *CC* calf circumference; *OR* odds ratio; *aOR* adjusted odds ratio; *CI* confidence interval; *NIHSS* National Institutes of Health Stroke Scale; *LOS* length of hospital stay

## Discussion

This study aimed to investigate the relationship between sarcopenia, possible sarcopenia, muscle mass, muscle strength, and CC, and the functional outcomes 3 months after stroke. Based on the results of this study, it was determined that the sole factor associated with functional prognosis 3 months after stroke is a decline in muscle strength.

The findings provide several important insights. First, the prevalence of sarcopenia in patients with acute stroke was 28%, consistent with previous studies highlighting the high prevalence of sarcopenia in this population [16, 20]. Second, all assessments, including sarcopenia, possible sarcopenia, muscle weakness, low muscle mass, and low CC, were significantly associated with functional outcomes 3 months after the stroke. These findings support the existing literature that recognises sarcopenia as an important prognostic factor for functional recovery in patients with stroke [17, 18, 29]. However, after adjusting for potential confounding factors, sarcopenia, muscle weakness, and low SMI were found to be independently associated with poor outcomes. This result aligns with previous studies [17, 18, 36–40], which have consistently emphasized the importance of assessing sarcopenia, muscle weakness, or muscle wasting as a prognostic factor in stroke patients. What our study newly revealed is that muscle weakness showed the strongest association with functional outcomes among these three assessments. It is worth noting that previous studies assessed functional outcomes only at hospital discharge, whereas our study specifically examined functional outcomes at 3 months post-stroke. These results suggest the importance of assessing muscle function even in environments where measurement tools are unavailable and it is not possible to measure skeletal muscle mass when predicting functional outcomes in patients with stroke.

CC measurement offers several advantages, including its non-invasive nature and ease of application, making it feasible for use in various clinical settings [27, 29]. However, our study did not demonstrate significant independent effects of low CC or possible sarcopenia, which included CC measurement, on functional outcomes in patients with stroke. Although CC has been proposed as a practical assessment tool for sarcopenia and for predicting functional outcomes in patients with stroke [27, 29], our results suggest that it may not be a strong predictor of functional outcomes at 3 months after stroke in this specific population. The selection of cutoff values for low CC in this study was based on the criteria outlined by the AWGS2019, which recommends < 34 cm for men and < 33 cm for women [12]. These cutoff values have been used in a previous study on sarcopenia and have shown associations with adverse health outcomes in older participants [41]. However, it is important to note that different cutoff values have been proposed in studies that specifically focused on patients with stroke [27–30]. The use of different cutoff values to define low CC could potentially affect the results and interpretation of the study findings. Currently, there is no consensus on the specific cutoff value for SRS assessments, including CC, in the context of stroke. Therefore, caution should be exercised when generalising the results of this study to populations using different cutoff values for low CC in patients with stroke.

This study has some limitations. First, the study population comprised patients admitted to a single institution, which could not include large subjects, and may have limited the generalisability of the findings. Future studies involving larger and more diverse populations are required to conduct multivariate analysis to include more covariate and to confirm these findings. Second, this study focused on functional outcomes at 3 months after stroke. Long-term follow-up would provide valuable insights into the relationship between sarcopenia and functional recovery over time. This study did not assess the accuracy of predictive models for poor outcomes in patients with stroke [42]. Although this study explored the association between sarcopenia assessment and functional outcomes, further research is needed to validate and assess the accuracy of these predictive models.

## Conclusion

In conclusion, this study offers robust evidence for an association between sarcopenia, muscle weakness, and low muscle mass and unfavourable functional outcomes 3 months after stroke. Particularly, muscle weakness exhibited the strongest association with outcomes among them. These insights underscore the imperative to prioritize the assessment of muscle weakness over other assessments in the acute stroke setting to optimize patient outcomes and care.

## Data Availability

The datasets analysed during the current study are available from the corresponding author on reasonable request.
